# The pediatric rheumatology objective structured clinical examination: progressing from a homegrown effort toward a reliable and valid national formative assessment

**DOI:** 10.1186/s12969-019-0308-7

**Published:** 2019-02-08

**Authors:** Megan L. Curran, Emma E. Martin, Erin C. Thomas, Rashmi Singh, Saima Armana, Asnia Kauser, Eesha A. Zaheer, David D. Sherry

**Affiliations:** 1 University of Colorado School of Medicine, Children’s Hospital of Colorado, 13123 E 16th Avenue, Box 311, Aurora, CO 80045 USA; 2 Ann & Robert H. Lurie Children’s Hospital of Chicago, 225 E. Chicago Avenue, Box 50, Chicago, IL 60611 USA; 3 Division of Rheumatology, Perelman School of Medicine, University of Pennsylvania, Children’s Hospital of Philadelphia, 3401 Civic Center Blvd., Philadelphia, PA 19104 USA

**Keywords:** Medical education, Objective structured clinical examination, Simulation, Fellowship education, Assessment, Program evaluation

## Abstract

**Background:**

Of 37 pediatric rheumatology fellowship training programs in the United States, many have three or fewer fellows at a given time, making large-scale assessment of fellow performance difficult. An objective structured clinical examination (OSCE) is a scenario-based simulation method that assesses individual performance, thus indirectly measuring training program effectiveness. This study describes the development and implementation of two national pediatric rheumatology OSCEs and methods used for programmatic improvement.

**Methods:**

OSCEs for pediatric rheumatology fellows were held in 2009 and 2011 during national rheumatology meetings using scenarios and assessment forms originally developed by a fellowship program director. The seven scenarios tested medical knowledge, physical exam and interpersonal skills. Pediatric rheumatologist evaluators assessed fellows’ performance using checklists and gave immediate feedback. Program directors were sent summaries of their fellows’ performances. Fellows evaluated the OSCE, providing organizational and scenario improvement suggestions. Programmatic changes to the 2011 OSCE were based on 2009 performance data and program evaluation feedback.

**Results:**

Twenty-two fellows participated in 2009 and 19 in 2011. Performance scores in similar scenarios did not change considerably over the two iterations. In 2009, 85.7% of participants reported desire to change clinical behavior. Assessors’ 2009 program evaluation data prompted changes in rating scales and removal of invalid or unreliable assessments. Negative evaluation data about individual stations decreased from 60% in 2009 to 15.4% in 2011. Fellows’ ratings of the experience’s overall value were similar in 2009 and 2011. The average experience ratings were lower among fellows who proposed scenario-specific improvements and higher among those who recommended organizational improvements.

**Conclusions:**

The 2011 examination exhibited programmatic improvement via reduction in fellows’ scenario-specific negative feedback. Fellows’ overall satisfaction did not change. Further work in scenario selection, assessment validation and inter-rater reliability will improve future pediatric rheumatology OSCEs.

## Background

Pediatric rheumatology is a small subspecialty in the U.S. with only 37 accredited three-year fellowship programs in 2018 and 90 active trainees in 2016 [1, 2]. There are fewer applicants than available positions. In recent years, 37–41 spots have been available nationally with 11–20 spots unfilled in the match per year [3]. Many programs have three or fewer fellows of all training levels in a given calendar year.

There is no national standardized curriculum for pediatric rheumatology training, but the American Board of Pediatrics (ABP) and the Accreditation Council for Graduate Medical Education (ACGME) mandate content that is to be taught [4, 5]. Additionally, fellows are expected to meet milestone levels for pediatric competencies within the six ACGME domains of competency [6, 7]; programs must report this data to the ACGME. Since the entirety of a fellow’s training is completed at one center, it is difficult for faculty to assess fellows on a large scale, including how their fellows perform in comparison to other programs, which would encourage improved teaching if deficiencies were noted.

Training programs use various methods of assessing learner performance, including multiple choice in-training examinations, end-of-rotation assessments by supervising physicians, direct observation of clinical skills and simulations. There are no published data about what and how assessments are used within pediatric rheumatology fellowships. Patwardhan, et al. showed in 2014 that training experiences are quite variable between programs including numbers of patients seen, procedures performed, call schedules and conference presentations. The authors call for standardization in training practices and increased hands-on training exams including objective structured clinical examinations (OSCEs) [8].

An OSCE is an educational exercise where trainees perform simulated scenarios, rated by assessors using validated checklists of knowledge and skills required to successfully complete the scenarios. Since 1975, OSCEs have helped postgraduate medical education program directors assess learner competency in an objective manner [9]. Trainees’ overall medical knowledge does not always correlate with other essential abilities like interpersonal skills and professionalism, which can be difficult to evaluate in traditional, knowledge-based faculty assessments [10]. OSCEs also serve as important tools for teaching and measuring the effectiveness of training programs [11, 12]. To date, there are no reported pediatric OSCEs at national subspecialty meetings, though regional pediatric gastrointestinal adult rheumatology OSCEs have been published [13, 14].

We describe here the development and subsequent implementation of two informal national pediatric rheumatology OSCEs (PROSCEs). The PROSCEs had two aims: 1) to provide performance feedback to fellows and program directors and 2) to begin validating the exercise. We hypothesized that utilizing 2009 program evaluation data to make changes to scenarios would decrease negative feedback in 2011. We set two improvement goals: 1) improve scenarios so that the proportion of scenario-specific complaints would decrease and 2) increase the fellows’ average rating of the value of the experience.

## Methods

### Participants and locations

The PROSCEs were held during American College of Rheumatology (ACR) meetings in Philadelphia (2009) and Chicago (2011). All U.S. pediatric rheumatology program directors were invited to have their fellows participate for $75 per fellow. Registration was capped at 21 fellows. Attending pediatric rheumatologists, fellowship directors and other volunteers (nurses, medical students, patients and parents) were recruited to serve as assessors. Professional actors were not used. Volunteers were reimbursed $50–$75 for their time and transportation costs. Transportation was provided to all fellows and attending physicians from the ACR meeting to local pediatric rheumatology offices. Dinner was served for all participants and the experience lasted approximately four hours. Investigational review board approval was obtained for the 2011 PROSCE.

The PROSCE was performed in patient exam rooms in large clinic spaces where multiple adjacent rooms were available. For both PROSCEs, 21 fellows were split into three rotation groups (A, B and C) of seven fellows. Each rotation group was assigned to a corresponding set of stations (A, B, and C) consisting of seven scenarios run in separate exam rooms. Each group rotated through the stations independently so that each scenario was run simultaneously with three different fellows and three different groups of assessors and actors.

### Preparation

To establish inter-rater reliability, assessors for each scenario met by role (i.e. physicians, nurses, parents) during the pre-PROSCE dinner to discuss scenarios. In 2009, verbal instructions for discussion were provided. In 2011, organizers provided structured documents asking assessors to review the scenarios and checklists, compare feedback techniques, standardize expectations and identify important skills that all fellows should demonstrate.

Before the start of the exercise, fellows predicted their own performance. In 2009, they used 100 mm visual analog scales (VAS) to predict their communication, professionalism, and overall PROSCE performance, anchored as “poor” and “excellent”, with “average” in the middle. In 2011, fellows predicted communication, professionalism, and performance in six general skills, but not their overall PROSCE performance, using scales from 0-poor, novice, intermediate, advanced, expert-4.

### Scenarios and assessments

Scenarios and assessment checklists were originally written by one experienced fellowship program director (DS) and piloted over a few years at two institutions in the early 2000s. The materials were well-regarded by participating fellows and attendings. Table 1 gives brief descriptions of each scenario, including the types of assessors utilized. Of the seven scenarios for the 2009 PROSCE, five were slightly modified and two new scenarios were used in 2011.

**Table 1:** 2009 and 2011 Pediatric Rheumatology Objective Structured Clinical Examination Scenario Descriptions

Scenario Number	Description	2009 Assessors	2011 Assessors
1	Inform a parent of a child recently diagnosed with lupus about risks and benefits of glucocorticoid treatment	pediatric rheumatologist, parent^a^	pediatric rheumatologist, parent^a^
2	Prepare a joint injection for a patient with juvenile arthritis without a nurse present	pediatric rheumatologist	scenario not performed
3	Perform joint aspiration and injection using an orange model (in 2009, scenario included consenting parent for the procedure)	pediatric rheumatologist, parent^a^, patient^a^	pediatric rheumatologist, nurse^b^, parent^a^, patient^a^
4	Perform a complete musculoskeletal exam on a patient with juvenile arthritis	pediatric rheumatologist, parent^a^, patient^a^	pediatric rheumatologist, parent^a^, patient^a^
5	Discuss a child’s diagnosis of juvenile dermatomyositis with a parent	pediatric rheumatologist, parent^a^	pediatric rheumatologist, parent^a^
6	Give a brief lecture about juvenile idiopathic arthritis to medical trainees	pediatric rheumatologist	pediatric rheumatologist, medical student^c^
7	Write an appeal letter to an insurance company supporting the use of a biologic drug for a juvenile arthritis patient	pediatric rheumatologist	scenario not performed
8	Complete multiple-choice questions about gait and its diagnostic significance	scenario not performed	No assessors
9	Practice delivering bad news to parents of a child with joint pain determined to have cancer	scenario not performed	pediatric rheumatologist, parent^a^

^a^Roles of parents and patients were played by volunteers, either true patients with pediatric rheumatic diseases, parents of children with rheumatic diseases or lay-person adults. They were not formally trained standardized patient actors

^b^Volunteer registered nurses from the pediatric rheumatology clinic

^c^Volunteer medical students recruited by email

Scenarios were intended to simulate a clinical situation that pediatric rheumatology fellows encounter in practice and be completed in 15 min. Fellows were aware of time limits. Assessors provided immediate feedback for five additional minutes. For example, at one station, fellows performed a complete musculoskeletal exam on a real patient with juvenile idiopathic arthritis. Before the simulation, the assessor examined the child and noted abnormalities, allowing accurate assessment of the fellow’s findings. The checklist assessed whether certain joint sites and fibromyalgia tender points were examined. During the feedback session, assessors demonstrated techniques and abnormalities fellows missed and patients and parents commented on fellows’ bedside manner.

Assessors were stationed in rooms with forms to complete while each fellow rotated through. To avoid bias, faculty assessed groups of fellows that did not include fellows from their own institutions. Attending physician assessment forms consisted mainly of skill and behavior-based questions in checklist format. Forms for non-physician assessors covered topics such as patient education abilities and communication skills. All assessors rated fellows’ communication, professionalism and overall performance via the 100 mm VAS scales (2009) and 0–4-point scales (2011) described above. In 2009 but not 2011, after all seven fellows in a group rotated through the station, the physician assessor ranked their performance on the scenario (1 = best, 7 = worst). Full descriptions of each scenario, assessment forms and a complete list of steps undertaken to develop and carry out the PROSCE are available upon request.

### Post-PROSCE

In an anonymous written survey administered immediately after the last station, fellows answered open-ended questions about their favorite/best and least favorite/worst parts of the PROSCE, overall satisfaction with the process and suggestions for improvement. They rated the value of their PROSCE experience on a 100-point VAS from poor to excellent. Responses were qualitatively and quantitatively analyzed.

After the PROSCEs, checklist results were analyzed using basic statistics. Overall scores between fellowship training years were compared using student’s t-tests. Program directors were sent copies of their fellows’ assessment checklists; videos of their musculoskeletal exams; and a de-identified presentation of all fellows’ scores in professionalism, communication and overall performance on each scenario. An individualized key was included identifying only their fellows’ scores.

### Program revision for 2011

Certain traditional methods of OSCE development were not used, such as gathering an expert panel to review scenarios and checklists for content validity or using psychometric item analysis [15]. Instead, the Deming Institute’s Plan-Do-Study-Act (PDSA) cycle framework was drawn upon when changing scenarios from 2009 to 2011 [16]. During the 2009 PROSCE, assessors evaluated the materials in real time. Participant program evaluation data regarding content and construct validity was then used to revise the exercise for 2011.

## Results

### Participants

Twenty-one spots were available for sign-up each year. In 2009, two fellows from the host institution shared a slot, participating as examinees in a few stations and filling in as actors in other stations. Nineteen fellows from other institutions representing all three training years and one international trainee participated. The PROSCE accommodated 21/87 (24%) of all U.S. pediatric rheumatology fellows in 2009 and 19/86 (22%) in 2011. One fellow completed the PROSCE both years. Including all assessors and actors, there were 38 additional participants in 2009 and 45 in 2011.

### Program revision for 2011

Scenario changes were made based on 2009 fellows’ performance, satisfaction, and suggestions for improvement. For example, many fellows performed poorly per the checklist specifications in the syringe preparation and joint injection stations. Some fellows had not yet learned injection skills and others were critical of assessors accepting only one technique as correct because techniques are different across institutions. In 2011, the syringe preparation station was removed and the injection station focused on techniques less subject to training variation. The checklists evaluated the six ACGME competencies at varying frequencies, falling under thirteen different competency elements (Table 2).

**Table 2:** Number of Cases Evaluating ACGME Competency Elements in the 2009 and 2011 Pediatric Rheumatology Objective Structured Clinical Examinations

ACGME Competency	Competency Elements	Number of Cases 2009	Number of Cases 2011
Patient Care: Procedural	Joint injection	2	1
Patient Care: Non-Procedural	Musculoskeletal exam	1	1
	Develop management plans	2	3
	Diagnostic decisions	1	3
Professionalism	Informed consent	2	2
	Demonstrate empathy	4	5
Interpersonal Communication	Relationship development	4	5
	Teaching skills	1	1
Systems-Based Practice	Letter to insurance company	1	0
Medical Knowledge	Medication side effects	2	3
	Disease knowledge	3	3
Practice-Based Learning	Patient education	4	5
	Resident education	1	1

Changes were also made to assessment methods. When scoring communication, professionalism and overall performance, 2009 assessors felt that using 100 mm VAS scales anchored poor to excellent was subject to a wide range of interpretation. 2011 scales were changed to reflect the Dreyfus developmental model of skill acquisition [17], anchored as 0-poor, novice, intermediate, advanced, expert-4. Scale changes complicated data comparisons between the two years. For this manuscript, 2009 VAS scores (0–100) were converted to the 5-point scale (0–4). Additionally, 2009 assessors said that ranking fellows’ performances from best to worst within one station was not an accurate method of assessing overall performance, so fellows were not ranked in 2011.

### Fellow assessment

Performance data collected during the two PROSCEs is not directly comparable across years; even when the same scenario was used, fellows, assessors and rating scales were different. Average fellow communication, professionalism and overall performance scores for each scenario as judged by the pediatric rheumatologist assessors are presented in Table 3. There were 20 to 50 additional assessment points per scenario checklist. The tendency of fellows to miss certain items was compelling and surprising to program directors. Table 4 presents a subset, providing insight into common errors of omission.

**Table 3:** Average Fellow Performance Ratings by Pediatric Rheumatologist Assessors Using a Five-Point Scale (0–4)* by Scenario

Scenario	Overall Performance		Professionalism		Communication	
	2009^a^	2011	2009^a^	2011	2009^a^	2011
1: Glucocorticoid explanation	3.08	2.68	2.75	3.74	2.69	3.21
2: Joint injection preparation	2.72	N/A	N/A	N/A	N/A	N/A
3: Joint injection simulation	2.59	3.00	3.24	3.42	2.98	3.11
4: Musculoskeletal exam	2.67	3.21	3.32	3.68	3.23	3.37
5: Dermatomyositis diagnosis discussion	3.13	2.61	3.42	3.17	3.14	2.89
6: Juvenile arthritis lecture	3.02	2.82	N/A	N/A	N/A	N/A
7: Insurance appeal	2.61	N/A	N/A	N/A	N/A	N/A
8: Gait analysis	N/A	Score not averaged	N/A	Score not averaged	N/A	Score not averaged
9: Bad news delivery	N/A	2.89	N/A	3.39	N/A	2.72

^a^2009 VAS scores (0–100) with anchors of poor and excellent were scaled to 2011 five-point scale from 0-poor, novice, intermediate, advanced, expert-4

**Table 4:** Selected Results from Scenario Checklists in the 2009 and 2011 Pediatric Rheumatology Objective Structured Clinical Examinations

Scenario	2009	2011
1: Gluco-corticoid explanation	10/21 did not mention risk of adrenal suppression; 6/21 did not say the risk-benefit ratio favored taking steroids	16/19 did not mention risk of adrenal suppression; 4/19 did not say the risk-benefit ratio favored taking steroids
2: Joint injection preparation	11/21 did not prepare the injection sterilely; 12/21 did not buffer the lidocaine	Scenario not used
3: Joint injection explanation and simulation	All fellows were respectful of parent’s concerns; 13/21 did not mention skin hypopigmentation as a side effect	All fellows were respectful of parent’s concerns; 12/19 did not mention skin hypopigmentation as a side effect
4: Musculo-skeletal exam	Of all assessed joints, none was examined by all 21 fellows; 7/21 fellows did not check forefoot	The wrist was the only joint examined by all 19 fellows; 8/19 fellows did not check forefoot
5: Dermato-myositis diagnosis discussion	11/21 did not mention possibility of abdominal pain; 14/21 did not mention possible emotional consequences of JDM	12/18 did not mention possibility of abdominal pain; 14/18 did not mention safety precautions necessary due to muscle weakness
6: Juvenile arthritis lecture	9/21 did not mention limb growth abnormalities; 11/21 did not mention jaw disease related to oligoarticular juvenile arthritis	10/19 did not mention limb growth abnormalities; 13/19 did not mention jaw disease related to oligoarticular juvenile arthritis
7: Insurance appeal	10/21 did not cite a source correctly for the letter; 10/21 did not describe patient consequences of denied treatment	Scenario not used
8: Gait analysis	Scenario not used	Not scored
9: Bad news delivery	Scenario not used	All 18 fellows were respectful of the parent; 11/18 did not ask how much information the parent wanted to know about cancer

Fellows’ pre-PROSCE estimations of their abilities were lower than their actual performance scores in both 2009 and 2011. Fellows estimated their communication and professionalism skills to be below 2.5 on a five-point scale, when actual average scores exceeded 3. In 2009, fellows’ predicted mean overall performance VAS score was 50.8; the actual average was 70.8. In 2011, fellows did not predict their overall performance across scenarios. For both years, when scores for each fellowship year are averaged and the differences in means compared, there is a trend in improvement between first and second, second and third, and first and third year fellows. However, the only significant difference was between 1st and 3rd year performance in 2009 (Table 5).

**Table 5:** Comparison of mean overall performance scores by training year using student’s t-test

PROSCE Year	Training Year	Mean Overall Performance Score^a^	SD	Comparison	*p* value
2009	1 (*n* = 8)	2.69	0.40	Years 1 to 2	0.125
	2 (n = 8)	2.95	0.43	Years 2 to 3	0.134
	3+ (*n* = 6)	3.19	0.30	Years 1 to 3	0.016
2011	1 (*n* = 9)	2.82	0.48	Years 1 to 2	0.056
	2 (*n* = 5)	3.22	0.25	Years 2 to 3	0.460
	3 (n = 5)	3.24	0.39	Years 1 to 3	0.074

^a^2009 VAS scores (0–100) from poor and excellent were scaled to 2011 five-point scale from 0-poor, novice, intermediate, advanced, expert-4

### Program evaluation

In 2009, all 22 participants filled out the program evaluation survey. 85.7% reported that they would change some aspect of their clinical behavior after participating. The most prevalent response themes were desires to increase personal education, improve preparation before patient encounters, and enhance the quality of interactions with patient families. One fellow said “[I will] be more aware of terms I use talking with families,” and another wrote “I’m going to write out all the steps of a complete joint exam so I don’t forget anything. Embarrassment is a powerful motivator.” In free-text responses; one said, “Thank you for caring about our education,” and another said the PROSCE was “a bit stressful but extremely well done. Would definitely want to participate in the future.”

In 2011, 16/19 (84%) participants filled out the survey. One said that fellows were given “really excellent feedback on physical exam skills and how to evaluate a gait.” Another said the “direct feedback” was one of the best parts of the experience in addition to “meeting [assessors] from other institutions (networking).” Nine reported that the best parts of the experience were receiving immediate feedback and the opportunity to get perspectives from attending physicians outside their own fellowship program.

For both years, fellows comments were coded as positive or negative and as scenario-specific or organizational. Many of the 2009 organizational comments, such as the time of day of the PROSCE, were difficult to change, though in response to a complaint about the time allotted between scenarios, two breaks were added in 2011. Some 2009 scenario-specific complaints suggested concrete improvements and were rectified for 2011. The proportion of scenario-specific improvement suggestions decreased from 60% in 2009 to 15.4% in 2011. 35% of all 2009 program complaints were related specifically to the joint injection station compared to 3.6% of 2011 program complaints. Therefore, revising individual stations per 2009 evaluation data decreased scenario-specific suggestions for 2011, which was one of our improvement goals.

Fellows rated the overall value of the PROSCE experience as 75.9 in 2009 and 75.5 in 2011 on a VAS scale of 100, so our goal to improve this rating was not met. However, both groups rated the PROSCE highly and recognized valuable aspects of the experience. One 2011 fellow gave a negative evaluation about the general process and an overall experience rating much lower than others (21 on VAS) bringing the 2011 average down considerably. The average PROSCE experience ratings were lower among fellows giving scenario-specific improvement suggestions and higher among fellows offering suggestions relating to overall organization.

## Discussion

Our experience shows that running a national OSCE starting as a “homegrown effort” using scenarios and checklists developed by one expert and local volunteers is feasible. However, the time and effort required to plan an OSCE should not be underestimated. Financial support for administrative staff and for organizing physicians, some protection from clinical responsibilities during planning months was necessary.

While many OSCEs use standardized patients, our PROSCE used real patients for various roles. Past studies have suggested that volunteer patients perform well in simulated encounters and that children participating in these types of examinations have a good sense of interpersonal dynamics and can provide effective feedback, but ethical implications exist [18–21]. More investigation is needed to determine the best role for real patients in future iterations.

The PROSCEs were the first opportunity to compare clinical skills of pediatric rheumatology fellows across institutions and provide feedback to program directors. Although differences in training affected assessment results, almost all fellows agreed that the experience was valuable. We removed ranking as a performance measure in 2011: checklists and global rating scales are chief OSCE scoring rubrics, not comparison of fellow performance [15].

Fellow performance data beyond what is reported in Table 3 will not be published; while valuable to the fellows and program directors for formative assessment, it is not yet a formally valid and reliable measurement of a fellow’s overall abilities as a nascent pediatric rheumatologist. As a measure of validity, it is expected that fellows further in training would perform better on a skills exam, at least in terms of medical knowledge, but this was not shown in either year, perhaps because scores were more influenced by communication skills developed in residency than knowledge learned in fellowship.

Program directors appreciated the data received from this novel assessment method, particularly about the ACGME competencies. The Pediatric Milestones Project [7], a joint initiative of the ABP and the ACGME, was launched in 2012 after both of the PROSCEs were administered, so milestone feedback was not included in our program director reports. However, we used a developmental model (novice to expert) for rating fellows in 2011, similar to the manner in which milestones utilize a developmental ontogeny [17]. We retrospectively mapped specific skills to ACGME competencies, which allowed us to better examine the value of each scenario. If future licensing boards mandate directly observed performance examinations for summative assessment to ensure fellow competence at the end of training, the PROSCE will be a valuable preparatory exercise.

The next PROSCE is tentatively planned to be held in conjunction with the ACR’s Pediatric Rheumatology Symposium in 2020. The planning committee includes pediatric rheumatologists with formal training in medical education, fellowship directors and fellows from across the country. This committee’s work will be informed by medical education literature about performance based assessments and standardized patients [12, 15, 22–26].

Revisions will allow future performance data to be interpreted more meaningfully. Validation of scenarios and checklists will improve content, construct and face validity and ensure that stations test skills generally enforced across all programs. We will calculate psychometric statistics on previous performance data and develop more stringent guidelines to minimize bias and improve inter-rater reliability. Prior to the PROSCE, we will ask assessors to rate videos of fellows at various skill levels performing scenarios and provide scores from expert assessors to improve reliability. New content, including a scenario testing a fellow’s ability to accurately collect a patient’s history of joint pain will be added and fellows will be provided scenario information ahead of time. Checklist items will be mapped to specific competencies and milestones, keeping the novice-to-expert assessment scale. These changes will further assist program directors and clinical competency committees with global assessment and ACGME milestone reporting.

## Conclusions

In this report, we discuss the development of two iterations of a pediatric rheumatology OSCE in which fellows practiced skills and received immediate formative feedback. The PROSCE is invaluable for small fellowship programs that cannot organize an objective assessment on such a scale. Other subspecialties interested in designing a similar assessment can emulate this process. Although formal OSCE reliability and validity methods were not used, scenario content validity was increased in 2011 by modifications based on participant feedback. Improvement in scoring reliability was challenging to prove, but rater training was improved in 2011. The experience provided important practice and feedback for fellows and program directors while gathering suggestions for future iterations. We assert that improvement of scenario simulation exercises can occur in real time using plan-do-study-act cycles. Our long-term goal is to administer the PROSCE more frequently with ongoing programmatic improvement to further benefit pediatric rheumatology fellowship education as a whole.

## Abbreviations

ABP: American board of pediatrics
ACGME: Accreditation council for graduate medical education
ACR: American college of rheumatology
OSCE: Objective structured clinical examination
PDSA: Plan-do-study-act
PROSCE: Pediatric rheumatology objective structured clinical examination
VAS: Visual analog scale
